# Tropical Mosquito Assemblages Demonstrate ‘Textbook’ Annual Cycles

**DOI:** 10.1371/journal.pone.0008296

**Published:** 2009-12-14

**Authors:** Donald C. Franklin, Peter I. Whelan

**Affiliations:** 1 School for Environmental Research, Charles Darwin University, Darwin, Northern Territory, Australia; 2 Medical Entomology, Centre for Disease Control, NT Department of Health and Families, Darwin, Northern Territory, Australia; University of Leeds, United Kingdom

## Abstract

**Background:**

Annual biological rhythms are often depicted as predictably cyclic, but quantitative evaluations are few and rarely both cyclic and constant among years. In the monsoon tropics, the intense seasonality of rainfall frequently drives fluctuations in the populations of short-lived aquatic organisms. However, it is unclear how predictably assemblage composition will fluctuate because the intensity, onset and cessation of the wet season varies greatly among years.

**Methodology/Principal Findings:**

Adult mosquitoes were sampled using EVS suction traps baited with carbon dioxide around swamplands adjacent to the city of Darwin in northern Australia. Eleven sites were sampled weekly for five years, and one site weekly for 24 years, the sample of *c*. 1.4 million mosquitoes yielding 63 species. Mosquito abundance, species richness and diversity fluctuated seasonally, species richness being highly predictable. Ordination of assemblage composition demonstrated striking annual cycles that varied little from year to year. The mosquito assemblage was temporally structured by a succession of species peaks in abundance.

**Conclusion/Significance:**

Ordination provided strong visual representation of annual rhythms in assemblage composition and the means to evaluate variability among years. Because most mosquitoes breed in shallow freshwater which fluctuates with rainfall, we did not anticipate such repeatability; we conclude that mosquito assemblage composition appears adapted to predictable elements of the rainfall.

## Introduction

Annual rhythms are a fundamental biological response to the seasons. They arise when organisms time growth, reproduction and dispersal to make optimal use of predictable seasonal events [1], [2]. As an aggregate affect across many species, community (assemblage) structure may also fluctuate seasonally in a predictable manner [3]. However, environments are frequently unpredictable and complex biotic interactions may overwhelm rhythmic responses. Nevertheless, annual rhythms are often depicted and interpreted as cyclic and constant from year to year, but quantitative demonstrations are few and rarely both cyclic and repeatable.

Highly predictable variation in day-length underpins seasonality in temperate regions, but in the tropics day-length varies little and rainfall assumes a much greater role in defining seasons. However, rainfall is inherently less predictable than day-length. Most tropical regions experience a monsoonal climate characterised by intense seasonality of rainfall (summer wet – winter dry) [4], but paradoxically the monsoon tropics has the greatest annual variability in rainfall in the world [5]. This paradox has profound and unresolved implications for the region's biota. Whilst some biologists have emphasized flexibility in life histories and behaviour as adaptations to life in the monsoon tropics [6], [7], others have focussed on adaptive responses to the underlying seasonality [8], [9].

Organisms whose lifespan is less than the relevant environmental periodicity can respond rapidly to the onset and/or prolongation of favourable conditions regardless of their predictability [10]. Tropical mosquitoes undergo several to many generations per year and can reproduce prolifically [11]. As their larvae and pupae are obligately aquatic, and development is affected little by temperature variations encountered in tropical lowlands, they are ideal subjects with which to identify relationships between rainfall and abundance.

Here, we examine seasonal and among-year variation in the composition of mosquito assemblages from the monsoon tropics using an extensive long-term monitoring dataset, and demonstrate strikingly cyclic patterns that vary little from year to year regardless of among-year variation in rainfall.

## Methods

### Biology of Tropical Mosquitoes

Female mosquitoes can lay from 30–300 eggs per oviposition episode and may have a number of oviposition episodes, depending on age and food requirements. In the tropics, most species complete several to many generations per year, life cycles generally being completed in from 1.5–5 weeks [11]. Although development rates are sensitive to variations in temperature, the range of temperatures encountered in the lowland tropics drives variation in rates that is measured in days rather than months [12], [13]. Most tropical mosquitoes lack any dormancy mechanism, the major exception being the tribe Aedini (includes the genera *Aedes* and *Verrallina*), whose eggs can be resistant to desiccation for months or even years. Aedini eggs are laid on moist substrates and depend on subsequent immersion for hatching and subsequent larval development [14].

### Study Area and Mosquito Surveys

**Figure 1:**
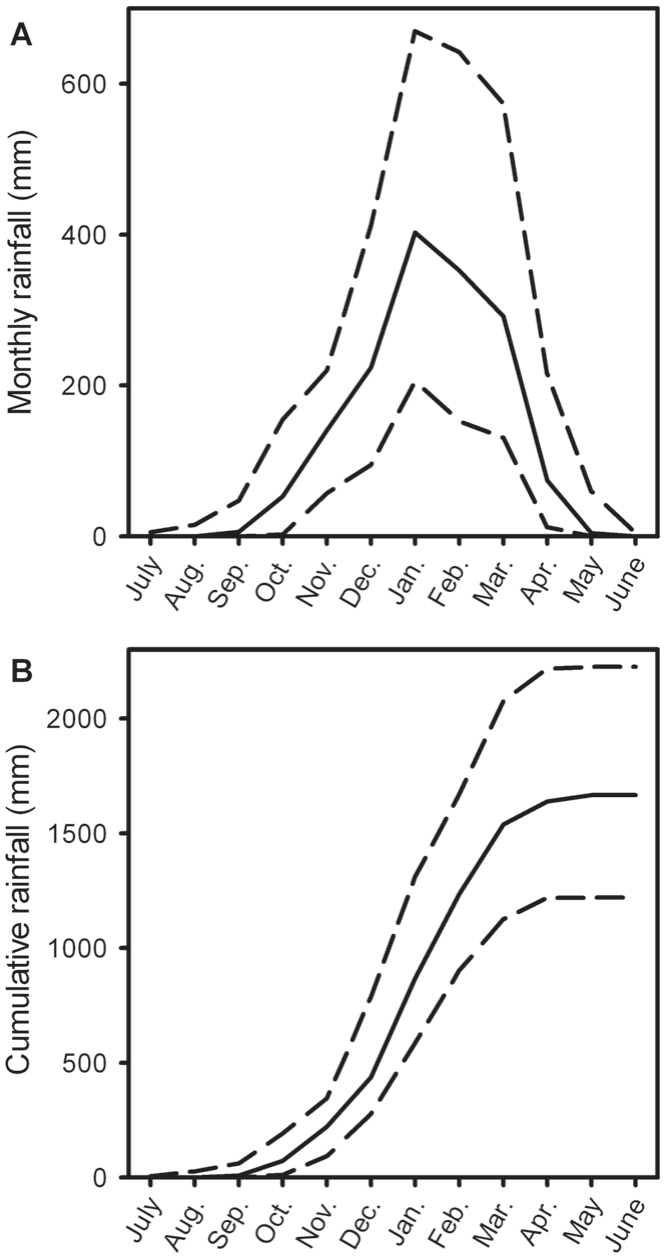
Seasonal pattern of rainfall for Darwin, northern Australia. Based on 68 years of data (1941–2008): (A) monthly; and (B) cumulative monthly calculated with actual years. Data are median ± 10^th^ and 90^th^ percentiles.

Mosquito populations were monitored at eleven woodland sites between the coastal city of Darwin (12°27′ S, 130°50′; Northern Territory, Australia) and the freshwater and tidal wetlands that almost surround the city [15]. With 88% of the mean annual rainfall of 1708 mm falling in the five ‘summer’ months (Nov. – March) at a mean rate of 9.9 mm d^−1^ (Figure 1a), the city's climate far exceeds the 55% and 3 mm d^−1^ thresholds employed to define a monsoonal climate [4]. Marked variation among years in rainfall, especially during the transitional months, affects the length of the ‘green’ season by up to three months [16], [17], but cumulative rainfall (Figure 1b) provides reliable annual saturation of soils.

Temperatures are high throughout the year. Mean monthly maxima range from 30°C in July to 33°C in October to December and in April, whilst mean monthly minima range from 19°C in July to 25°C from October to February [18]. In most years, overnight temperatures do not drop below 15°C. Day-length varies annually from 11.2 to 12.9 hours, with global radiation remaining relatively constant throughout the year because on the inverse seasonal relationship between cloud cover and potential solar radiation [18].

The number and identity of blood-seeking adult female mosquitoes were monitored overnight once per week at 11 sites for five years (2001 to 2005; 2,871 trap-nights), and weekly at one of these sites for 24 years (Palm Creek, 1982 to 2005; 1,253 trap-nights)), using suction traps baited with carbon dioxide. These were standard EVS CO_2_-baited mosquito traps [19] which detect a wide range of species [20]. Palm Creek was selected for long-term analysis because it offers the combination of long-term data with little human environmental change in its mosquito catchment area.

Samples were frozen after collection for later identification by the staff of Medical Entomology of the Department of Health and Families in Darwin. Samples of less than 300 individuals were identified and counted fully. For larger catches, the full catch and a sub-sample of *c*. 300 specimens were weighed and the sub-sample fully identified and enumerated. The sample was checked for species absent from the sub-sample. The ratio of weights provided a conversion factor to estimate the total number of individuals of each species in the sample. The abundance of a species present in the sample but absent from the sub-sample was scored as the total number present in the sample.

### Data Analysis

For both the 11-sites and Palm Creek datasets, weekly counts/estimates of the abundance of each species were averaged up to months (n = 60 and 288 months respectively), the former after combining sites into a single dataset. The resulting two datasets each comprise a matrix of species by months. For each month in the 11-sites data set, we summed the estimated number of mosquitoes and number of mosquito species, and calculated species diversity using Simpson's Diversity Index. This diversity index ranges from 0 (low diversity) to almost 1, a high diversity score indicating both greater species richness and relatively little variation among species in their abundance [21].

Each data set was then ordinated. Ordination is a form of data reduction applicable to multi-dimensional data sets. Assemblages or communities of species may be envisaged as points in an *n*-dimensional hypervolume in which each dimension represents the abundance of a species, and the Euclidean distance between pairs of points is a measure of the dissimilarity of those assemblages. In ordination, one seeks to reduce the number of dimensions for ease of interpretation – usually but not necessarily to one to three dimensions – whilst retaining as much of the original distance information as possible. Non-metric multidimensional scaling (MDS or NMDS) is a robust non-parametric form of ordination [22] with wide application to the analysis of species assemblages, including the analysis of change to ecological communities [23]. We chose MDS because our species assemblage abundance scores are strongly skewed by an abundance of zeros and thus not normally distributed. MDS has previously been used to represent annual cycles in assemblage composition [3], [24].

Computationally, the first step is to calculate a triangular matrix of dissimilarity among all pairs of samples. The Bray-Curtis distance measure is appropriate for assemblage data because it gives no weight to zero/zero (mutual absence) data. It ranges from 0 (identical) to 1 (no overlap), and is calculated as:
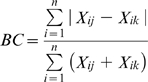
where *BC* = the Bray Curtis index of dissimilarity; *X_ij_, X_ik_* = the number of individuals of species *i* in the samples *j* and *k*; and *n* = the number of species in the samples [21]. MDS then seeks solutions for pre-determined levels of dimensionality whilst miminising stress using iterative selection of steepest descent in stress. Stress is “a measure of difference in monotonicity in the relationship between” the original and reduced dimensional dissimilarity matrices [25], and is thus greatest when the number of dimensions is least. Solution axes provide relative scaling of final dissimilarity but are otherwise arbitrary. The cloud of points may thus be rotated around their centroid (the mean of axis scores), allowing one to align maximum linear variance in the ordination with an axis for further consideration – a process known as varimax rotation.

For the purpose of ordination, mosquito species present in fewer than four samples were excluded, leaving 43 species in each dataset [26]. Monthly species data were ln(x+1)-transformed to reduce the overwhelming effect of a few abundant species. Ordination was undertaken in the software PC-ORD 4.01 [25] using the Bray-Curtis distance measure and MDS. Four hundred iterations were allowed to generate minimum-stress solutions. Outputs were evaluated in from 1 to 6 dimensions and the optimal dimensionality identified as that in which the stress was significantly (*P*<0.05) better than random and in which the addition of another dimension reduced ordination stress only slightly. The 11-site ordination was repeated with different random starting coordinates to check for robustness. The solutions presented have been subject to varimax rotation.

To identify the species influencing ordination structure, a biplot vector for each species was fitted through the ordination centroid [25]. Ordination points (months) are scored for the point on the line to which they are perpendicular, and the correlation between this position and the species' abundance calculated. The vector is rotated around the ordination centroid to optimise the correlation. For each species, the optimal correlation was evaluated for significance (*P*<0.05) after application of the sequential Bonferroni correction for the number of species in the ordination.

Species represented by a significant biplot vector in the 11-site ordination were characterised for breeding site ecological attributes (salinity: brackish/salt *cf* freshwater; waterbody type: temporary pools *cf* containers *cf* permanent or semi-permanent waterbodies). The angle of biplot vectors was used as a measure of the seasonal peak of each species, and mean seasonal peaks of attribute groups evaluated with Watson-Williams (circular) *F*-tests. Species belonging to more than one attribute group were excluded from analysis. Post-hoc evaluations of waterbody types were pair-wise with a Bonferroni correction.

## Results

**Figure 2:**
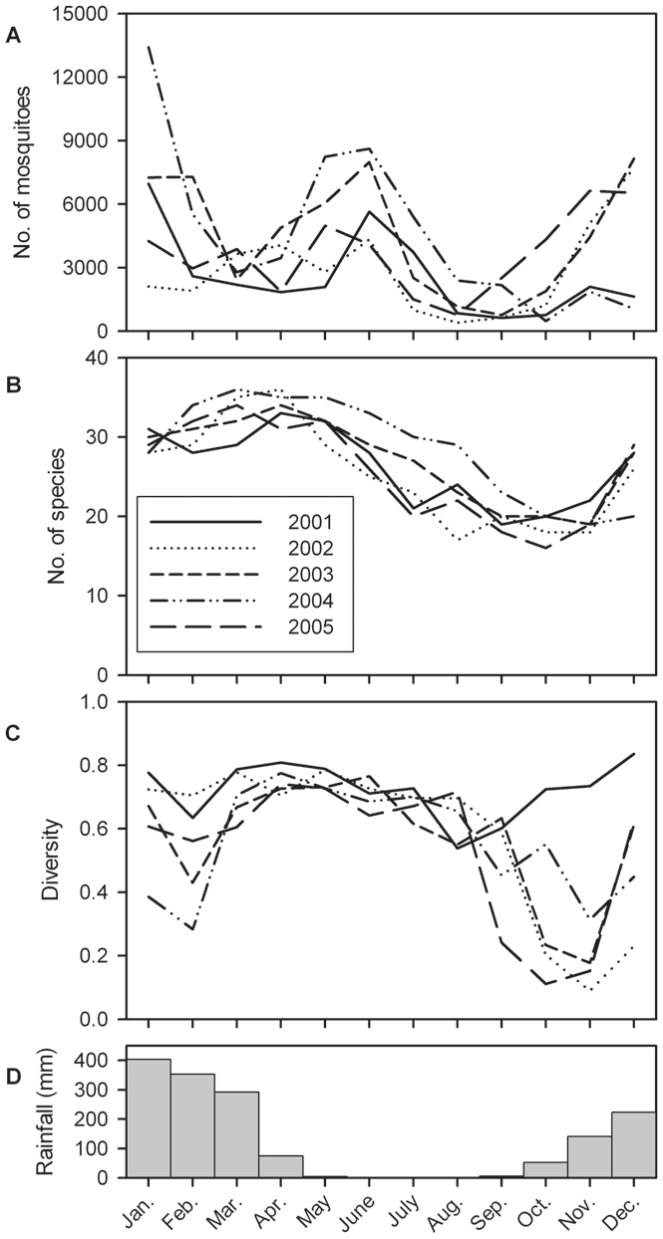
Trends in mosquito abundance over five years. Based on weekly sampling of 11 sites around the city of Darwin: (A) weekly average number of individuals; (B) total number of species; and (C) species diversity (Simpson's Diversity Index). Median monthly rainfall (68 years) is shown in (D) for comparison.

Five years of data from 11 trap sites yielded 56 species from an estimated 617,272 mosquitoes, of which 334,291 (54%) were identified. In some years, mosquito abundance peaked early in the wet season and again early in the dry season, but in all years was low later in the wet season and later in the dry season (Figure 2a). Species richness varied predictably with time of year, peaking in the wet-dry transition (April – 33.8 species±1.92 SD) and bottoming in the dry-wet transition (October – 18.8 species±1.79 SD) (Figure 2b). Diversity was consistently high from late in the wet season (March) to mid-late dry season (August) but highly variable in the dry-wet transtion and early to middle wet season (Figure 2c), the latter reflecting the propensity for a few species to establish vast numerical preponderance at these times.

**Figure 3:**
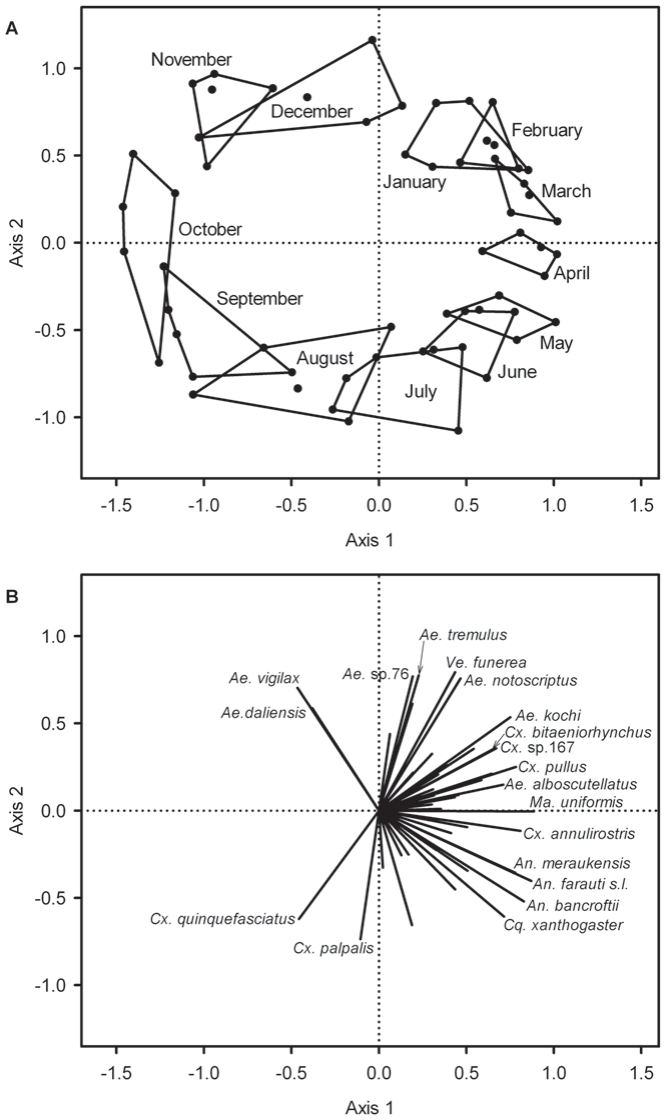
Ordination (A) and biplot (B) of mosquito samples. Based on the abundance of 43 species at 11 sites combined, with samples in common months (n = 5 years) linked to form convex polygons. (B) comprises significant (Bonferroni-corrected) biplot vectors for species (only those with *r*>0.7 are labelled). The length of vectors in (b) is relative to their *r* value, scaled to the axes coordinates. *Ae.* = *Aedes*; *An*. = *Anopheles*; *Cq*. = *Coquillettidia*; *Cx*. = *Culex*; *Ma*. = *Mansonia*; *Ve*. = *Verrallina*.

A two-dimensional ordination solution (Figure 3a) was optimal and robust (stress = 7.8; random stress = 31.5), with repeat ordination yielding an identical result. The solution retained 96.4% of the original variance (axis 1–72.3%; axis 2–24.1%). Calendar months were non-randomly grouped (Mantel randomisation test, *P* = 0.001) and demonstrated a striking annual cycle with little variation among years. All species included displayed significant (*P*<0.05 with sequential Bonferroni correction) linear radial correlations with the 5-year ordination (Figure 3b). Most species peaked between January and June. Brackish/saltwater specialists peaked earlier in the wet season than freshwater specialists (Watson-Williams *F* = 15.3, d.f = 36, *P*<0.001). Species that breed in transient pools or containers peaked earlier in the wet season than those of permanent or semi-permanent waterbodies (Watson-Williams *F* = 12.3, d.f. = 2,35, *P*<0.001).

**Figure 4:**
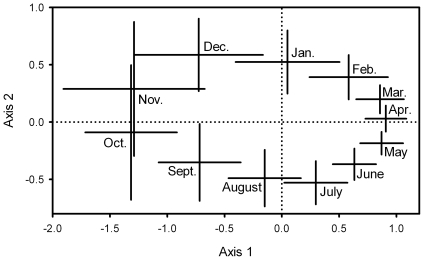
Ordination of 24 years of mosquito samples. Based on the monthly abundance of 43 species at Palm Creek. Data are monthly centroids ± one standard deviation.

At Palm Creek, an estimated 788,840 mosquitoes were collected, of which 38.3% (302,895) were identified, also comprising 56 species. A two-dimensional ordination solution (Figure 4) was optimal and robust (stress = 12.5; random stress = 32.1). The solution retained 92.5% of the original variance (axis 1–79.2%; axis 2–13.3%). Calendar months were non-randomly grouped (Mantel randomisation test, *P*<0.001). Variation among years was markedly greater than for the 11-sites (5 year) ordination but summary of axis coordinates (mean ± standard deviation) nevertheless retrieved a clear and similar annual cycle.

Both ordinations illustrate that the composition of the mosquito assemblage was most predictable at the end of the wet season and least so at the end of the dry season, aligned with Axis 1, and that the greatest annual contrasts in assemblage composition were between these times.

## Discussion

Ordination of these mosquito assemblages has depicted strikingly predictable annual cycles. These were driven by a succession of species peaks in abundance and predictable seasonal variation in species richness. Notwithstanding the intensely monsoonal climate, many species persisted as adults throughout the year, probably by continuous breeding in refugia as for *Culex* and *Anopheles* species [27]. Predictable cycles occurred notwithstanding the irruptive tendencies of a few species [28], [29]. We acknowledge minimizing the impact of irruptive tendencies on our ordinations by logarithmic transformation of abundance, but note that fundamental changes in community compostion (e.g. rare species becomes common or *vice versa*) will still strongly influence ordination position. We believe that irruptions by a few medically-important species have clouded perceptions of system predictability. Our data show that major mosquito irruptions occurred with the cessation and especially the onset of the wet season – and are thus in themselves relatively predictable in timing – and that they do not substantially influence overall assemblage composition.

Ordination has been recommended for the analysis of temporal change in community structure [23]. Quantitative measures of variation among years may be derived from locations in ordination space, for example as area of the minimum convex polygon (Figure 3a), as measures of variance in axis scores (Figure 4), or with the variety of more sophisticated methods for calculating area or volume developed for the analysis of faunal home ranges [30]. Ordinations of time series of the composition of other species assemblages have yielded less striking and/or less repeatable annual cycles; the best we could identify being for fish and crustaceans monitored monthly for four years in a Florida estuary [24]. Other interesting examples are for the fish fauna of an estuary in Belgium [31], and for crustaceans in a Spanish estuary [3]. In none of these cases, nor any other that we have located, was among-year variation quantified.

It is doubtless no coincidence that the above examples are all estuarine. The marine environment is relatively buffered against short-term and unpredictable variation in environmental conditions. Deep freshwater aquatic environments may also be so buffered but shallow and riparian habitats, such as where most mosquitoes breed, are highly responsive to rainfall, especially in the extreme climate of the monsoonal tropics. The mosquitoes in our study mostly breed in shallow (<200 mm) freshwater, with a few species breeding in deeper water to 1 m associated with aquatic or semi-aquatic vegetation, and some in tidally influenced environments. It is surprising, therefore, that the Darwin mosquito assemblage displayed such organised and repeatable temporal structure. The predictable seasonality of the monsoon is the overwhelming influence on assemblage composition. We interpret the seasonal succession of species in this study as indicating fine environmental partitioning adapted to predictable rainfall elements [1], a product of the diversity of niches available [32] and possibly a long ecological and evolutionary history [9].

Predictable annual cycles may be anticipated in environments where assemblages are structured directly by seasonal, tidal or daylight rhythms. Ironically but most usefully, they may provide sensitive indicators of changing climates [33] because they are relatively insensitive to short-term climatic aberrations.
